# Meat grinder hand injury; case report

**DOI:** 10.1016/j.ijscr.2022.106768

**Published:** 2022-01-17

**Authors:** Hassan Salad IBRAHIM, Engin ILKER, Huseiyn TASKOPARAN

**Affiliations:** Mogadishu Somali Turkish Research and Training Hospital, Mogadishu, Somalia

**Keywords:** Meat grinder, Hand injuries, Metal cutting saw, Amputation, Microsurgery

## Abstract

**Introduction and importance:**

Meat grinder injury to the hand is not a common case but can cause a wide spectrum of injuries that are difficult to treat. Management of meat grinder hand injuries is complex and should be based on a clear understanding of principles of wound management, fracture fixation, and soft-tissue reconstruction. To our knowledge, here we reported the first case of meat grinder hand injury faced by a practicing surgeon in an underdeveloped country (Somalia) with a Successful outcome.

**Case presentation:**

A 2-year-old boy had his right hand trapped in a meat grinder. The middle and ring fingers were severely crushed at the metacarpophalangeal joint. Under general anesthesia, all digits were reconstructed.

**Clinical discussion:**

Meat grinder hand injuries can result in a wide range of injuries, from simple fractures to amputations. Safe extraction of the hand is considered the most important determinant of outcomes. Preoperative antibiotics, wound irrigation, and microsurgical techniques are used in the treatment.

**Conclusion:**

Although treating meat grinder hand injuries is challenging, careful extraction of the hand with a metal cutting circular saw will not only prevent secondary damage but will also save time.

## Highlights

• Meat grinder hand injuries are complex injuries, that result in tremendous physical and emotional impact.
• The most important aspect in the treatment is the safe removal of the trapped hand from the grinder without causing further damage.
• metal cutting circular saw to extract the hand is considered to be safer compared to reverse spin of the meat grinder.

## 1 Introduction

Meat grinder Occupational accidents frequently result in hand injuries. Negligence and inexperience are the main factors in these incidents. In addition to significant economic effects, it causes widespread trauma resulting in tremendous physical and emotional distress. Especially when it occurs in children the treatment has a long-term impact on the family [1]. As a result, a surgeon or a team dealing with mutilating hand injuries must be aware of the surgical principles to provide the best possible care [2]. In Somalia, where such surgical teams are rare, the results are not always satisfactory. We present our experience of a 2 years old child with a meat grinder hand injury. This case report has been reported in line with the SCARE Criteria [3].

## 2 Case report

**Fig. 1:**
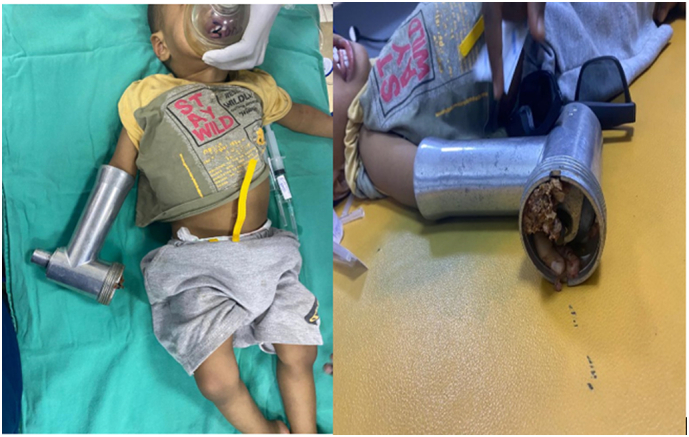
The right hand trapped inside the meat grinder, causing obvious finger crushing.

**Fig. 2:**
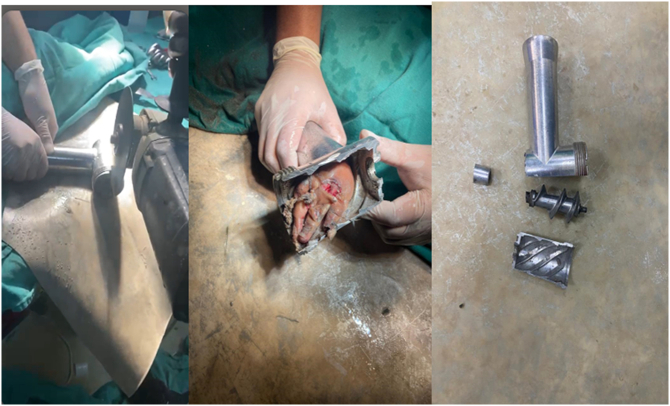
Metal cutting circular saw was used to free the hand.

**Fig. 3:**
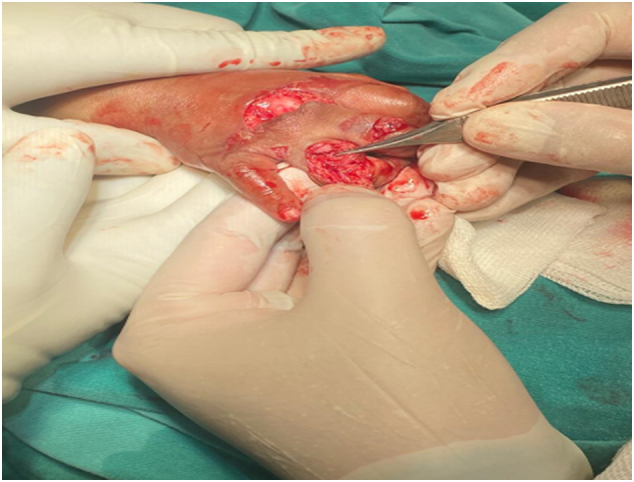
Dorsal view of the hand after debridement and irrigation.

**Fig. 4:**
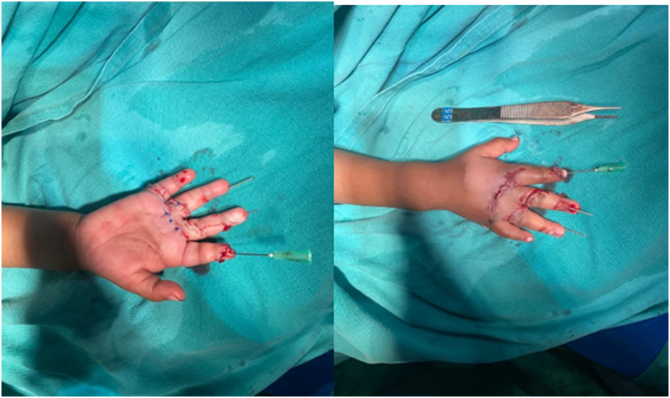
Immediate postoperative dorsal and volar view of the hand.

**Fig. 5:**
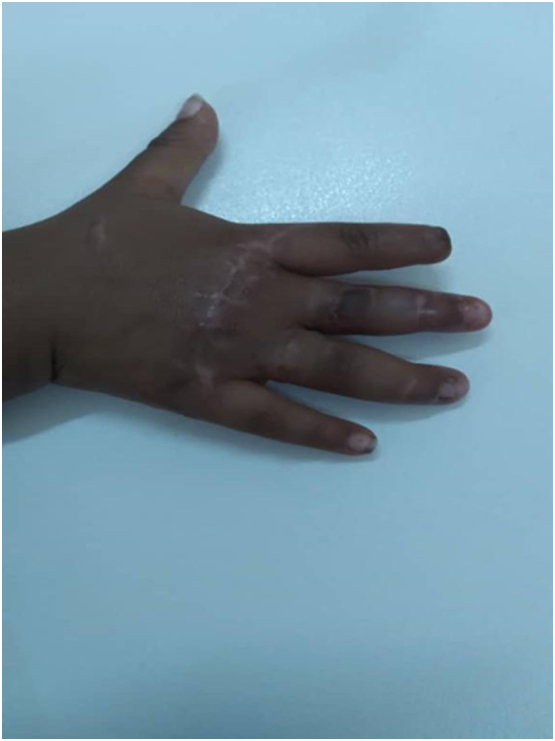
Postoperative one month view of the hand.

A 2-year-old boy patient was brought to the emergency room by his father, with his right hand trapped in a meat grinder automatic machine in October 2020 (Fig. 1). There was no history of psychiatric illness or drug use. His mother noticed what happened only after the child screamed and turned off the grinder. After pain medication administered in the emergency department extrication of his hand was not possible without further damage. Immediately the patient was taken to the operation theatre, under general anesthesia the meat grinder machine was sliced into two parts with the use of a metal cutting circular saw, and the hand was freed (Fig. 2). There was no active bleeding noticed, the middle and ring finger were severely crushed at the metacarpophalangeal joint (MCP)and proximal interphalangeal joint (PIP) (Fig. 3). The decision to salvage all digits was made, wounds were thoroughly irrigated and debrided. The 2nd, 3rd, as well as the 4th digits were fixed with k wires. The 4th digit's extensor tendon was also repaired. The wound was sutured using absorbable rapid vicryl. Postoperative final appearance of the hand was satisfying (Fig. 4). After two weeks of antibiotics, analgesics, and regular wound care, the patient's functional hand was restored without any disability (Fig. 5).

### 2.1 Operator

Dr. Hassan Salad, Orthopedic, and Trauma Senior Resident at Mogadishu Somali Turkish Training and Research Hospital, under the supervision of Dr. Engin Ilker, Orthopedic and Trauma consultant. The operation was done at Mogadishu Somali Turkish Training and Research Hospital in Mogadishu, Somalia.

### 2.2 Patient perspective

The patient's family was satisfied with the overall intervention and outcome.

## 3 Discussion

Our hands are an integral part of our existence. They are not only crucial for physical and psychological growth but also for our appearances and professional careers [4]. Meat grinder hand injuries are uncommon, but when they do occur, they cause gruesome and possibly irreversible injuries in young active patients [5]. Because the outcome of these injuries is dependent on the knowledge of the first surgeon, Mistreatment or delay in treatment of these injuries might cause the hand to get so mangled that even the most competent surgeon will be unable to extract anything from it which will have a long-term psychosocial impact on the patient [6].

The most important aspect in the treatment of these injuries is the safe removal of the trapped hand from the grinder without causing further damage. The most typical way used to extract the hand in the literature review is to reverse spin the grinder using an adjustable wrench or Rongeur until the damaged hand is freed [1], [4], [5], [7]. In our case, a metal cutting circular saw was used to cut the meat grinder into two parts, normal saline was used to keep to saw from overheating, an osteotome was inserted just above hand within the meat grinder to prevent additional injury to the hand, this technique is less time consuming and less likely to inflict further hand injuries.

Following hand extraction, an attempt should be made to restore an acceptable hand (one with three fingers near-normal length, near normal PIP joint motion, and good sensibility, as well as a functioning thumb) [2].

To repair a mutilated hand, meticulous debridement, wound irrigation, microsurgical techniques for reimplantation, and fracture fixation are all essential. Even though plastic or hand surgeons were not available in our context, we were able to achieve an acceptable outcome for the patient. This case is unique among the reported cases of such a condition because all digits were preserved.

## 4 Conclusion

Effective management of meat grinder hand injuries is largely dependent on the time consumed and the technique used to extract the hand without causing additional trauma. We believe that a metal cutting circular saw is an ideal tool.

## Consent

Written informed consent was obtained from the patient's parent for publication of this case report and accompanying images. A copy of the written consent is available for review by the Editor-in-Chief of this journal on request.

## Provenance and peer review

Not commissioned, externally peer-reviewed.

## Ethical approval

Ethical approval was not needed for writing a case report in our settings.

## Funding

The authors received no funding from any individual or institution, and this work is completely voluntary.

## Research registration number

Not applicable.

## Guarantor

Hassan Salad Ibrahim.

## CRediT authorship contribution statement

Hassan Salad Ibrahim was involved in study design, data acquisition, drafting the article, revising it critically, and finally approved the manuscript.

## Declaration of competing interest

The authors report no conflict of interest of any sort.
